# Novel Endoscopic Closure of Iatrogenic Hypopharyngeal Perforations Using Through-the-Scope Tissue Approximation Clips

**DOI:** 10.14309/crj.0000000000002215

**Published:** 2026-07-03

**Authors:** Pranav Ramamurthy, Kais Zakharia

**Affiliations:** 1 Department of Internal Medicine, University of Massachusetts Chan Medical School – Baystate Medical Center, Springfield, MA; 2 Division of Gastroenterology, University of Massachusetts Chan Medical School – Baystate Medical Center, Springfield, MA

**Keywords:** hypopharyngeal perforation, endoscopic closure, through-the-scope clips, iatrogenic injury, upper esophageal sphincter

## Abstract

Hypopharyngeal perforation is a rare but potentially life-threatening complication of upper aerodigestive tract instrumentation. Management strategies remain controversial, and data supporting flexible endoscopic closure in this region are limited. We describe 3 cases of iatrogenic hypopharyngeal perforations successfully managed with MANTIS through-the-scope tissue approximation clips as part of a multidisciplinary approach. All defects were closed endoscopically. Two patients achieved complete mucosal healing without surgical intervention. One patient experienced clinical decline related to comorbid illness rather than closure failure. Endoscopic closure using through-the-scope clips may be a feasible option in carefully selected patients when performed early with multidisciplinary collaboration.

## INTRODUCTION

Perforation of the upper aerodigestive tract is an uncommon but serious complication associated with significant morbidity and mortality, particularly when diagnosis or intervention is delayed.

Although thoracic esophageal perforations have been extensively studied, hypopharyngeal and cervical esophageal perforations are rare and lack standardized management guidelines.^1,2^

Traditionally, hypopharyngeal perforations have been managed surgically with transcervical exploration and drainage, rigid endoscopic repair, or conservative therapy depending on defect size, timing of diagnosis, and clinical stability.^2,3^ With advances in endoscopic closure techniques, minimally invasive approaches are increasingly used for gastrointestinal perforations. However, reports describing flexible endoscopic closure in the hypopharynx remain limited.^4–7^

The MANTIS clip (Boston Scientific, Marlborough, MA), a reopenable and rotatable through-the-scope tissue approximation clip, is designed for large-defect closure using anchor prongs to grasp tissue edges. We present 3 cases of iatrogenic hypopharyngeal perforations successfully managed using MANTIS through-the-scope clips.

## CASE REPORT

This retrospective case series includes 3 patients treated at a single tertiary referral center between 2023 and 2025. Patients were diagnosed with suspected or confirmed iatrogenic hypopharyngeal perforation during or shortly after transesophageal echocardiography (TEE) or endoscopic retrograde cholangiopancreatography (ERCP). Decisions to pursue endoscopic closure were made collaboratively with otorhinolaryngology and surgical teams. Clinical data were obtained from the institutional electronic medical record.

### Case 1

**Figure 1.**
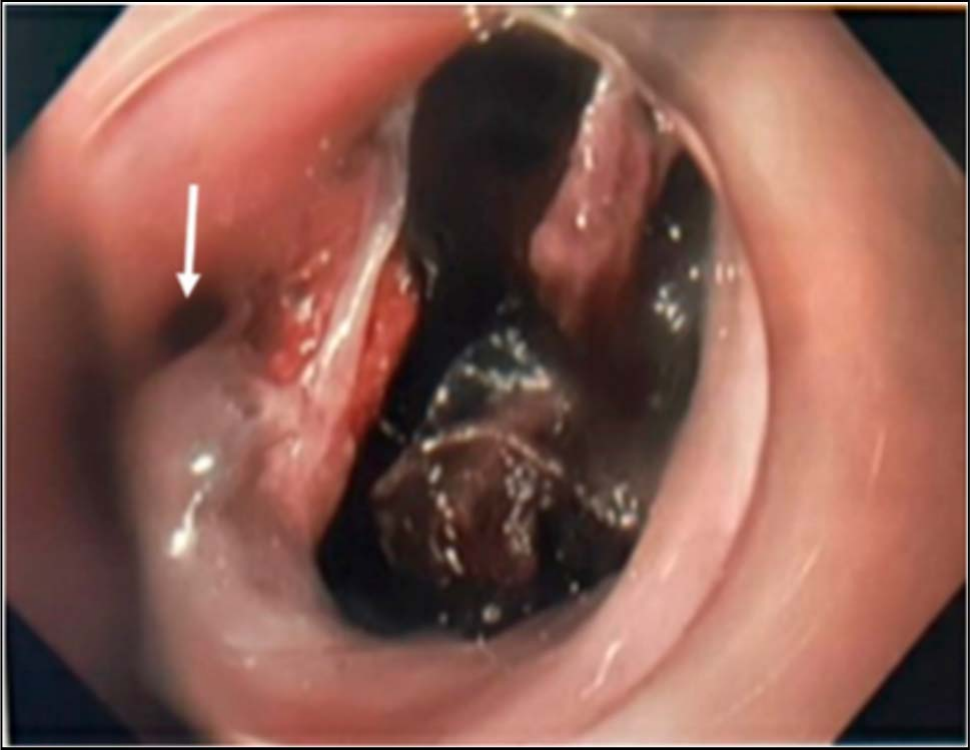
Endoscopic view of full-thickness hypopharyngeal perforation immediately proximal to upper esophageal sphincter in Case 1. The defect is located along the posterior hypopharyngeal wall with visible disruption of the mucosa and exposed underlying tissue. Esophageal lumen denoted by white arrow.

An 84-year-old woman underwent aortic valve replacement and ascending aortic aneurysm repair complicated by gastrointestinal bleeding shortly after intraoperative TEE. She developed hemodynamic instability requiring vasopressors and transfusion. The patient was taken emergently overnight within 12 hours of TEE for gastroenterology and otorhinolaryngology intervention. Esophagogastroduodenoscopy (EGD) with a standard 9.9 mm diameter 103 cm working length gastroscope with distal attachment cap revealed a full-thickness hypopharyngeal perforation immediately proximal to the upper esophageal sphincter (UES) (Figure 1).

**Figure 2.**
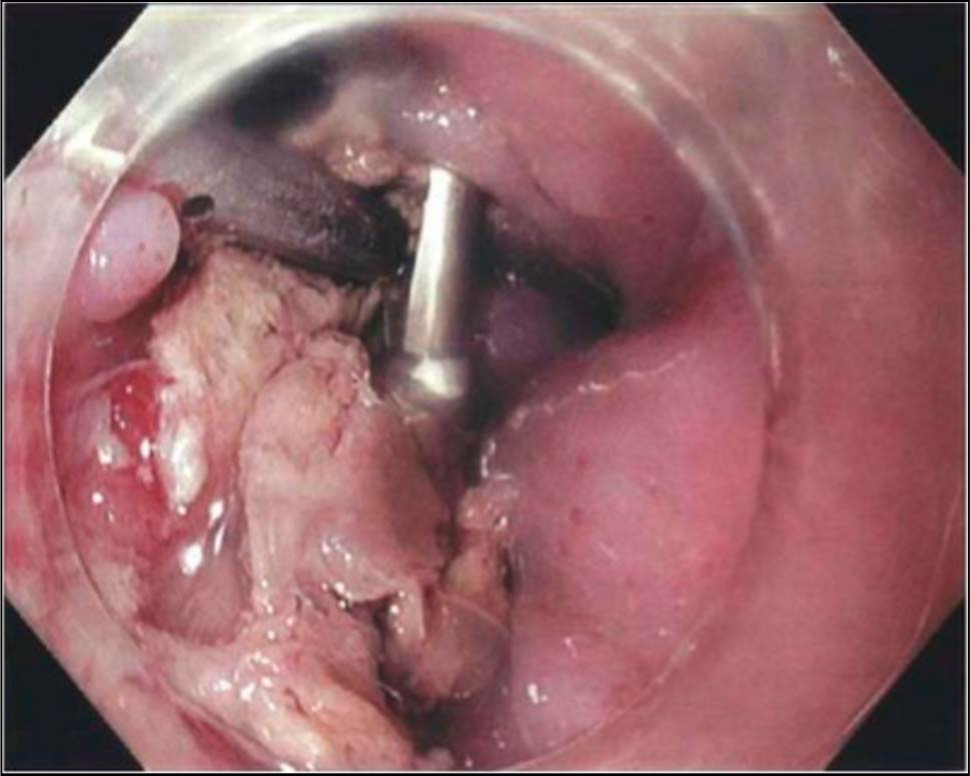
Endoscopic view of repaired full-thickness hypopharyngeal perforation following deployment of 4 MANTIS through-the-scope tissue approximation clips in Case 1. Two clips achieved effective grasping of opposing wound edges, resulting in partial closure of the hypopharyngeal perforation with improved tissue apposition.

Given high surgical risk, flexible endoscopic closure was performed. Considering the size of the defect, it was felt that traditional short-stem through-the-scope clips would be insufficient to provide sufficient tissue approximation and defect closure. Over-the-scope clips were also considered but it was felt that these would be inappropriate given the limited maneuvering space within the hypopharynx. Four MANTIS through-the-scope clips were deployed, with 2 achieving effective tissue approximation (Figure 2). Otorhinolaryngology placed oropharyngeal packing and a transcervical drain.

**Figure 3.**
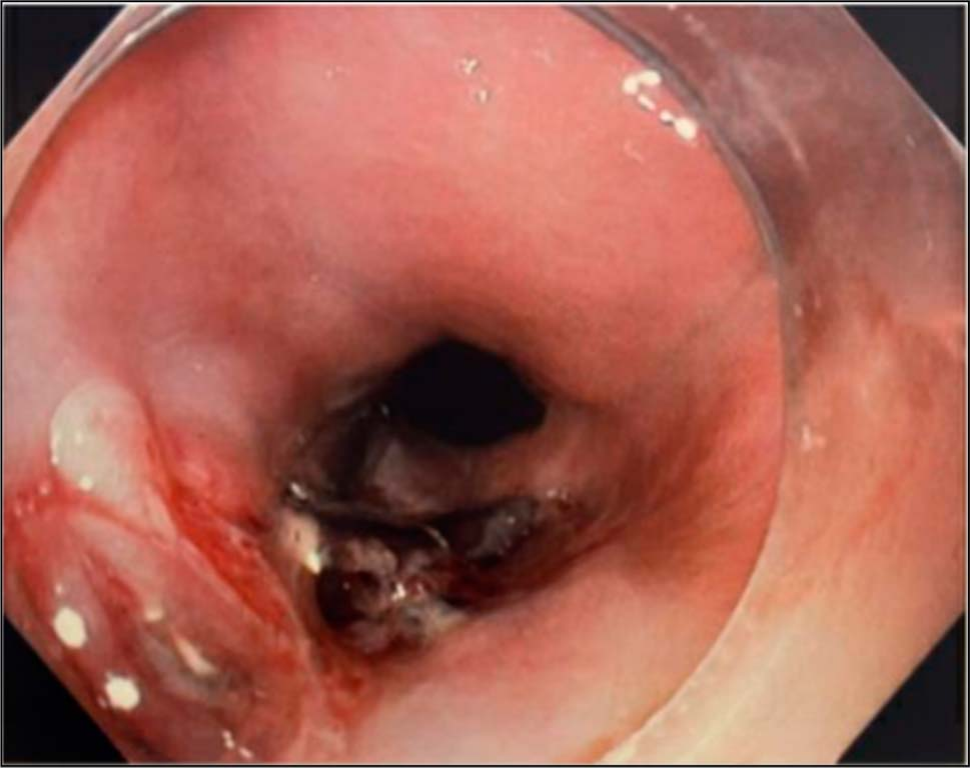
Follow-up esophagogastroduodenoscopy at 1 month in Case 1 demonstrating interval healing of the previous hypopharyngeal perforation site, with re-epithelialization and no visible residual defect or evidence of persistent leak. Two MANTIS clips noted to remain in-situ.

**Figure 4.**
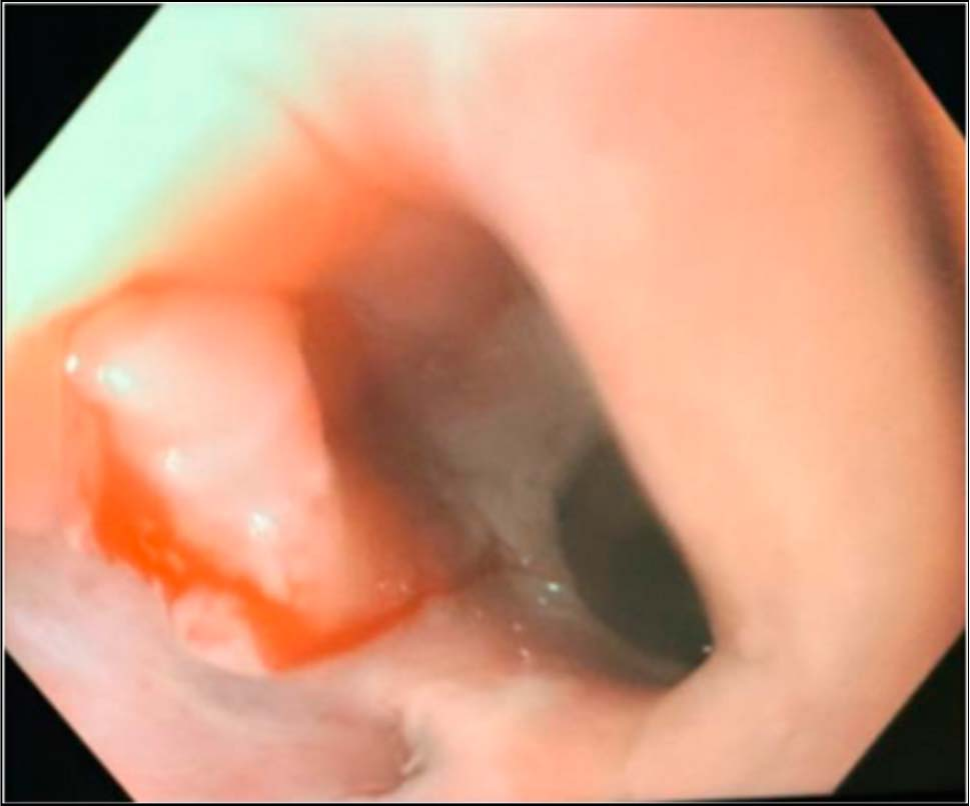
Endoscopic view following removal of retained MANTIS clips approximately 6 weeks after initial placement in Case 1. The mucosa at the former perforation site appears well healed without dehiscence or active bleeding.

The patient was started on total parenteral nutrition within 2 days and was maintained on broad-spectrum antibiotics. She later underwent tracheostomy and surgical gastrojejunostomy tube insertion about 10 days after initial EGD due to persistent oropharyngeal dysphagia in the setting of repaired perforation and postcritical illness dysphagia with subsequent initiation of enteral feeding. Follow-up EGD at 1 month demonstrated healing without persistent leak (Figure 3). The MANTIS clips were removed at 6 weeks (Figure 4). The patient returned to baseline swallowing function 8 weeks after clip removal, as demonstrated by modified barium swallow. Computed tomography scan of the neck with IV contrast demonstrated no acute abnormalities. The patient continued on a regular diet and gastrojejunostomy tube was eventually removed about 10 weeks after initial insertion.

### Case 2

**Figure 5.**
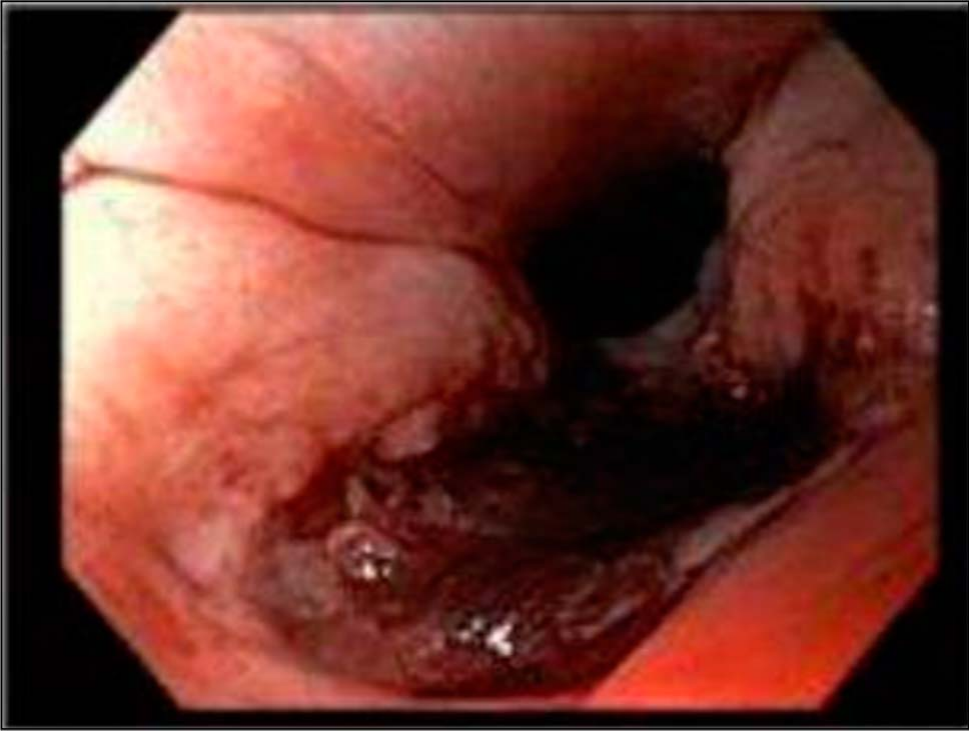
Endoscopic view in Case 2 showing a large mucosal defect of approximately 2.5 cm with adherent clot formation located just proximal to the upper esophageal sphincter after difficult duodenoscope passage during endoscopic retrograde cholangiopancreatography. The appearance is concerning for deep hypopharyngeal injury.

A 78-year-old woman with gallstone pancreatitis and choledocholithiasis underwent ERCP with a 11.3 mm duodenoscope with 13.5 mm distal end and 124 cm working length. Resistance was encountered at the hypopharynx. The duodenoscope was immediately withdrawn and a standard 9.9 mm diameter 103 cm working length gastroscope with distal attachment cap was inserted which revealed a large mucosal defect with clot proximal to the UES (Figure 5). Of note, there was no subcutaneous crepitus in the neck and the insufflation was adequate which suggested against full-thickness perforation. Decision was undertaken to pursue endoscopic closure. A guidewire was advanced to the stomach and the side-viewing duodenoscope was advanced through the wire successfully into the duodenum under direct visualization following which biliary sphincterotomy and stone extraction were performed. The duodenoscope was retracted and the standard gastroscope was once again inserted.

**Figure 6.**
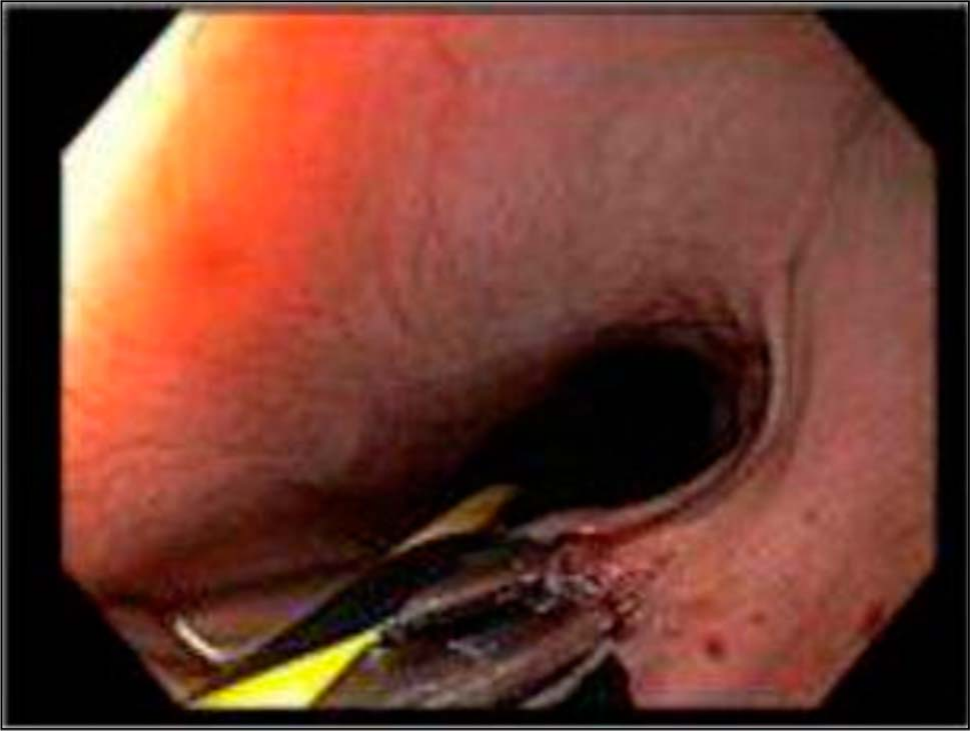
Endoscopic image after deployment of 2 MANTIS clips in Case 2 demonstrating complete approximation of hypopharyngeal defect with secure closure and restored mucosal continuity.

**Figure 7.**
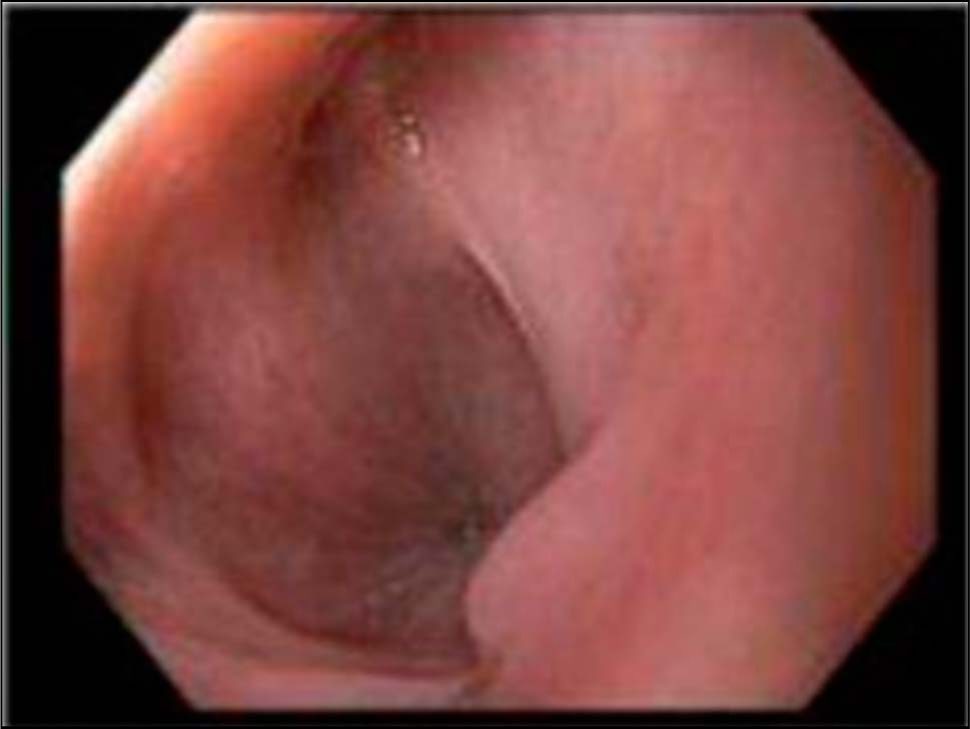
Surveillance endoscopy at 3 months in Case 2 demonstrating complete mucosal healing at the prior perforation site. No residual defect is visible, and previously placed clips have spontaneously detached.

Two MANTIS through-the-scope clips were deployed with complete closure (Figure 6). A nasogastric tube was placed under visualization. She underwent laparoscopic cholecystectomy the following day after which total parenteral nutrition was initiated. Follow-up barium esophagram at 4 days showed no cervical emphysema or pneumomediastinum. Repeat EGD 5 days following index procedure confirmed closure of a 2.5-cm defect. Enteral feeding via nasogastric tube began 10 days after the index procedure. The patient returned to baseline within 5 weeks. Surveillance endoscopy at 3 months showed complete mucosal healing with spontaneous clip detachment (Figure 7).

### Case 3

A 92-year-old woman with choledocholithiasis and *Escherichia coli* bacteremia was transferred after suspected perforation during ERCP at an outside hospital. Contrast-enhanced computed tomography demonstrated trace pneumomediastinum and pneumothorax without fluid collections. She required intubation and vasopressor support.

**Figure 8.**
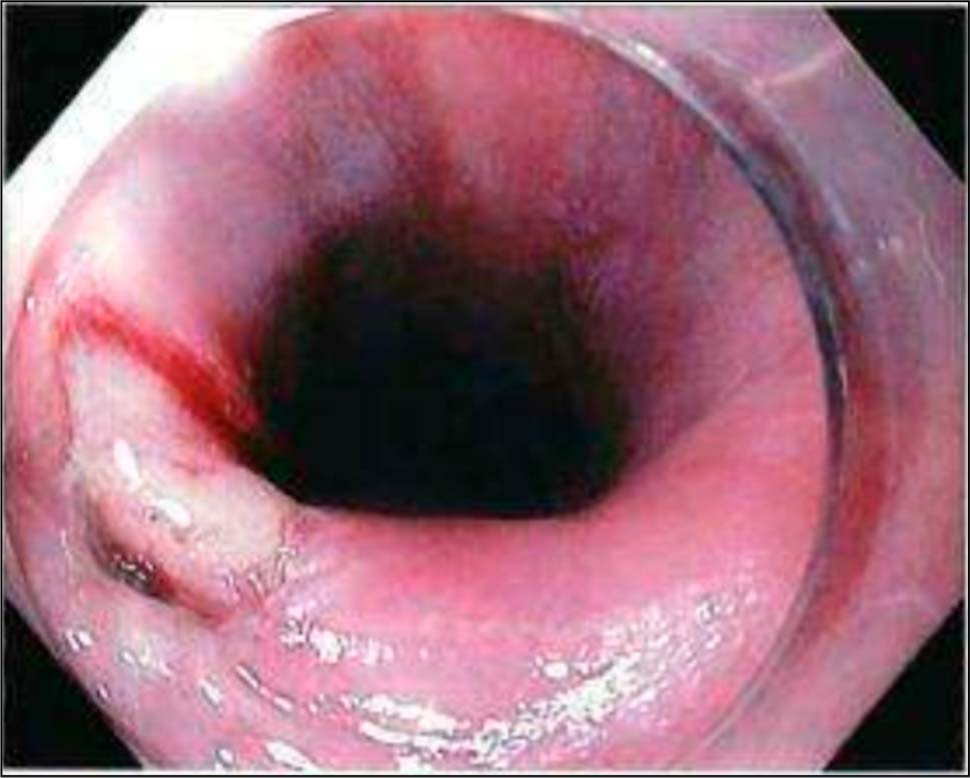
Endoscopic view in Case 3 demonstrating a 1 cm full-thickness perforation at the level of the upper esophageal sphincter with clear visualization of disrupted mucosa and exposed deeper layers.

**Figure 9.**
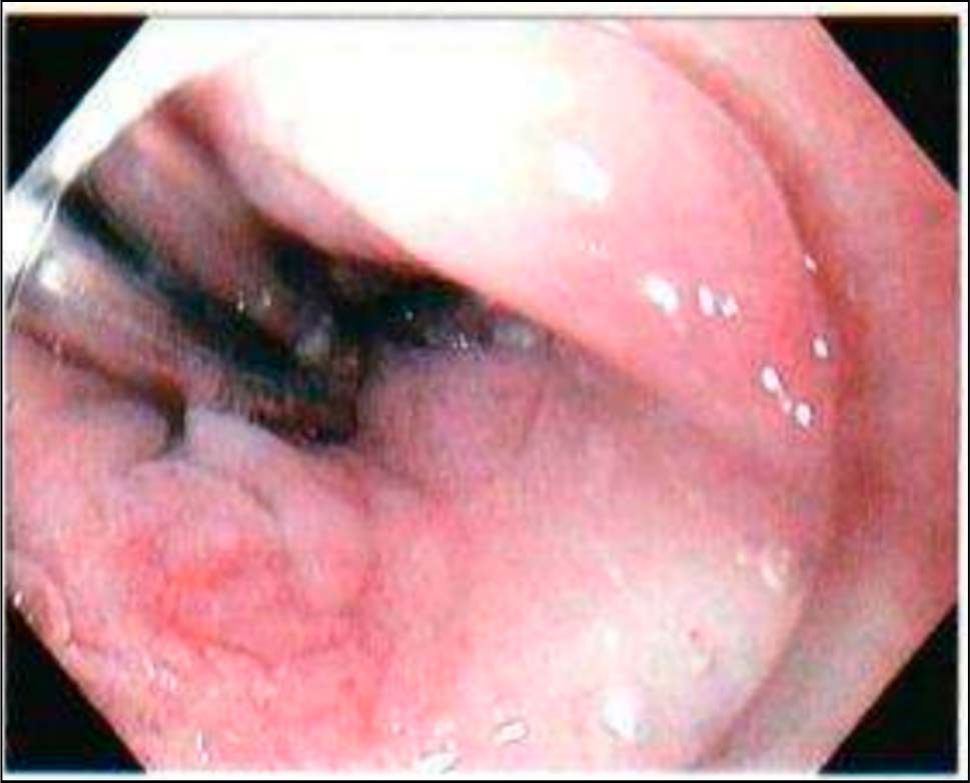
Endoscopic view after placement of 4 MANTIS clips in Case 3 showing excellent tissue approximation and complete closure of the hypopharyngeal perforation with adequate alignment of wound edges.

EGD with a standard 9.9 mm diameter 103 cm working length gastroscope with distal attachment cap demonstrated a 1-cm full-thickness perforation at the UES (Figure 8). Four MANTIS through-the-scope clips were deployed with complete closure of the defect (Figure 9). A nasogastric tube was placed and the patient was maintained on broad-spectrum antibiotics and antifungals. The patient stabilized and left the intensive care unit after being started on total parenteral nutrition within 2 days. Despite technical success, her course was complicated by progressive respiratory failure and pleural effusions in the setting of pulmonary thromboembolism with right heart failure and atrial fibrillation with rapid ventricular response. Care was transitioned to comfort measures within 1 week of admission, and follow-up endoscopy was not performed.

## DISCUSSION

Hypopharyngeal perforation is rare but high risk, with reported mortality rates up to 20%–40%, largely driven by delayed diagnosis and mediastinitis.^1–3^ Early recognition and prompt intervention remain the most important determinants of outcome.^3^

Endoscopic closure has emerged as an effective strategy for esophageal perforations, with success rates exceeding 80% in selected cases.^4,5^ Current expert guidance supports endoscopic therapy for iatrogenic perforations when feasible, emphasizing early intervention and multidisciplinary management.^6,7^ However, application in the hypopharynx has been limited by anatomic constraints and airway concerns.

A previous report described combined use of over-the-scope and through-the-scope clips for cricopharyngeal injury during peroral endoscopic myotomy.^8^ By contrast, our series demonstrates successful repair using MANTIS through-the-scope clips alone. In all 3 cases, MANTIS clips achieved effective tissue approximation without airway compromise or need for surgical salvage. The rotatable and reopenable design of the MANTIS clip facilitated precise tissue capture in the confined working space of the hypopharynx.

A review of other flexible endoscopic repair methods reveals that over-the-scope clips, fully covered self-expandable metal stents and endoscopic vacuum therapy have also been described for the repair of hypopharyngeal perforations in selected case reports and series.^9–12^ A comparison of these approaches is detailed in Table 1 and technical considerations regarding these approaches are described further on.

**Table 1.** Endoscopic management of hypopharyngeal perforations

Authors	Etiology	Size/location	Closure technique	Outcomes
Buscaglia et al.^10^	Iatrogenic (endoscopy)	Hypopharynx	Polyflex self-expandingplastic stent	Successful closure without surgery 1/1
Ribeiro Jordão Sassoet al.^9^	Foreign body perforation	Hypopharynx	Modified EVT	Successful healing; no stenosis 1/1
Hernández Mondragónet al.^8^	Iatrogenic(POEM-related tear)	Cricopharyngeal region	Mantis-like claw TTS clipplus conventional clips	Successful rescue closure
Loske et al.^12^	Iatrogenic	Cervical esophagus(4 patients)	EVT	Successful defect closure in 4/4
Current case series (2023–2025)	Iatrogenic (TEE, ERCP)	Hypopharynx proximalto UES; 1–2.5 cm	MANTIS TTS clips alone	Technical success 3/3; complete mucosal healing in 2; 1 death unrelated to leak; no surgical salvage

Summary of published reports and current case series describing endoscopic management of hypopharyngeal and proximal cervical esophageal perforations. Reported cases include predominantly iatrogenic and foreign body-related injuries involving the hypopharynx, cricopharyngeal region, or proximal esophagus. A range of endoscopic closure techniques were used, including self-expanding plastic stents, endoscopic vacuum therapy, and through-the-scope clipping systems, including MANTIS TTS clips. Outcomes across studies demonstrate high rates of technical and clinical success, with most cases achieving complete defect closure without the need for surgical intervention. The current case series highlights the feasibility of MANTIS TTS clips as a primary closure modality for hypopharyngeal perforations measuring up to 2.5 cm, with successful technical closure in all cases and no requirement for surgical salvage.

ERCP, endoscopic retrograde cholangiopancreatography; EVT, endoscopic vacuum therapy; POEM, per-oral endoscopic myotomy; TEE, transesophageal echocardiography; TTS, through-the-scope; UES, upper esophageal sphincter.

In our case series, 2 patients demonstrated complete mucosal healing with return to baseline swallowing function. The third patient experienced clinical decline attributable to cardiopulmonary comorbidities without radiographic or clinical evidence of persistent leak or mediastinitis. No patient required emergent surgical salvage for closure failure. Time to endoscopic closure was short in all 3 cases, with repair performed during the index procedure or within 24 hours of recognition. Early identification and prompt endoscopic intervention likely contributed substantially to favorable outcomes. These findings suggest that flexible endoscopic closure with MANTIS clips may be a viable alternative to surgery in selected patients when performed early within a multidisciplinary framework.

Adjunctive management is essential following flexible endoscopic closure with MANTIS clips and includes broad-spectrum antibiotics, strict nil per os status, nasogastric suctioning, airway monitoring, adequate analgesia, and nutritional support. This represents standard conservative therapy for esophageal perforation of any type and location. All patients were admitted to the intensive care unit postprocedure, with close airway monitoring. Nasogastric suctioning was initiated in all patients while maintaining nil per os status. Total parenteral nutrition was initiated in all cases followed by advancement to enteral feeding via nasogastric or gastrojejunal access when appropriate. Oral feeding was resumed only after radiographic or endoscopic confirmation of closure and close monitoring to rule out mediastinitis or failure of closure. Antibiotics were initiated as early as possible and continued for at least 7–10 days, covering both aerobic and anaerobic organisms. Antifungal coverage was also provided in Case 3 of our series, but this is not empirically recommended. Systemic analgesia was provided to all patients. Local anesthetic spray with topic phenol 1.4% was also available to help manage local irritation, pain or foreign body sensation though none of the 3 patients required its use. We would suggest all these interventions be routinely implemented for patients undergoing closure of hypopharyngeal perforations with MANTIS clips.

Technical considerations were critical given the confined anatomy. A standard gastroscope fitted with a distal attachment cap improved visualization and stability. The rotatable and reopenable design of the MANTIS clip allowed precise positioning before deployment, while anchor prongs facilitated secure tissue capture and minimized slippage in edematous mucosa. Sequential clip placement enabled progressive approximation of defect edges without airway compromise. By contrast, short-stem through-the-scope clips are limited in these larger defects due to limited jaw opening width and lower clamping force and their use would likely require a much greater number of clips with a higher risk of requiring salvage with MANTIS clips or other modalities.^8^

Over the scope clips offer strong tissue capture but require cap mounting that increases distal tip diameter and limits maneuverability in the hypopharynx. Stent placement has only been described in a single case abstract of malignancy-associated perforation and carries additional risks including stent migration and airway compression.^10^ Endoscopic vacuum therapy is effective in larger or delayed leaks but requires repeated exchanges and intensive airway management, especially in cervical esophageal perforations.^12^ In our cases, due to early recognition and intermediate to large defect size, closure with MANTIS clips was deemed to be an appropriate primary therapy, with alternate flexible or rigid endoscopic closure and surgical approaches reserved for salvage therapy.

Limitations include small sample size and retrospective design. Larger studies are needed to define patient selection criteria and long-term outcomes.

Flexible endoscopic closure using MANTIS through-the-scope tissue approximation clips is a feasible option for selected cases of iatrogenic hypopharyngeal perforation. Early diagnosis, airway protection, and multidisciplinary collaboration are essential for favorable outcomes.

## DISCLOSURES

Author contributions: P. Ramamurthy: Conceptualization, data acquisition, manuscript drafting, critical revision and final approval. K. Zakharia: Procedures, data interpretation, critical revision and final approval. P. Ramamurthy is the article guarantor, accepts full responsibility for the conduct of the study and the integrity of the data.

Financial disclosure: None to report.

Informed consent was obtained for this case report.

## ABBREVIATIONS:

EGD: esophagogastroduodenoscopy
ERCP: endoscopic retrograde cholangiopancreatography
TEE: transesophageal echocardiography
UES: upper esophageal sphincter
